# DrugBLIP: exploring the protein–molecule interaction mechanisms with a multi-task learning graph transformer

**DOI:** 10.1093/bioinformatics/btag069

**Published:** 2026-04-10

**Authors:** Rubo Wang, Xingyu Gao, Peilin Zhao

**Affiliations:** Institute of Microelectronics, Chinese Academy of Sciences, Beijing, 100029, China; University of Chinese Academy of Sciences, Beijing, 100049, China; Institute of Microelectronics, Chinese Academy of Sciences, Beijing, 100029, China; University of Chinese Academy of Sciences, Beijing, 100049, China; School of Artificial Intelligence, Shanghai Jiao Tong University, Shanghai, 200230, China

## Abstract

**Motivation:**

Traditional drug discovery methods are costly and inefficient, while existing deep learning approaches remain limited by task specificity and practical applicability. Accurately modeling protein–molecule interactions is critical for advancing virtual screening, docking, and drug design.

**Results:**

We propose DrugBLIP, a multi-task graph transformer model based on SE(3)-equivariant architectures, to unify protein–molecule interaction learning. By integrating contrastive learning, matching tasks, and docking optimization, DrugBLIP captures 3D spatial relationships through a hybrid graph transformer framework. Evaluations demonstrate state-of-the-art performance: DrugBLIP achieves an AUROC of 0.8217 and BEDROC of 0.5743 on virtual screening, outperforming traditional and deep learning baselines by 10%–127% across metrics. It also attains 91.2% top-1 docking success on CASF-2016 and 41.8% target fishing accuracy, showcasing robustness in diverse scenarios. Additionally, DrugBLIP reduces computational time by 700× compared to traditional docking tools.

**Availability and implementation:**

Code is available at https://github.com/Wolkenwandler/DrugBLIP and archived at Zenodo with DOI: 10.5281/zenodo.16990700.

## 1 Introduction

Drug discovery is one of the most challenging and innovative fields in medical science, playing a crucial role in safeguarding human health. According to statistics, the average cost of developing a new drug exceeds 2.6 billion dollars, and the success rate is <10% (Zhang *et al.* 2025), which underscores the substantial risk and investment required. However, with the continuous progress of technology, the methods and tools for drug discovery are becoming increasingly diverse, ranging from traditional drug screening to modern biotechnology. In recent years, the efficiency and success rate of drug discovery have shown measurable improvement (Paul *et al.* 2010). High-throughput screening technology enables researchers to quickly screen millions of compounds in search of potential drug candidates, while the application of computer-aided drug design and artificial intelligence has created new opportunities to enhance both accuracy and efficiency (Zhang *et al.* 2025).

Traditional scoring functions are integral to the entire drug design process, including virtual screening, docking, and scoring and ranking. However, traditional methods for docking and scoring protein pockets and ligand molecules primarily rely on force fields and empirically developed physical principles (Forli *et al.* 2016). These methods are highly dependent on experience and the limitations of simulated force fields, making them time-consuming and unsuitable for large-scale virtual screening. For instance, precise calculations of intermolecular interactions, such as van der Waals forces, electrostatic forces, and hydrogen bonds, are complex and time-intensive. Additionally, the accuracy of these methods is constrained by the parameters of the force fields used, leading to variability and increased uncertainty in results. In contrast, modern machine learning approaches can significantly reduce computation time and improve accuracy by learning from large datasets, making them more suitable for large-scale virtual screening. Therefore, integrating advanced data-driven approaches with existing strategies is a promising direction to overcome the inherent limitations of force field-based methods.

Inspired by the fact that multi-task training can achieve relatively good results in multiple downstream tasks (Li *et al.* 2022), we propose to unify the objectives of traditional scoring functions and investigate drug–protein interaction mechanisms through a multi-objective hybrid training framework, termed **DrugBLIP** (**Drug** action mechanism via **B**oosting **LI**gand–**P**rotein interaction), as a potential replacement for conventional scoring functions. Our method demonstrates competitive performance across diverse tasks, highlighting its potential as a robust alternative. Specifically, we constructed a pre-training dataset that captures the structural information of protein pockets and ligand molecules based on their structural matching and existing data (Wang *et al.* 2005, Francoeur *et al.* 2020). We pre-trained a model based on a SE(3) graph transformer on this dataset, allowing it to effectively learn 3D representations of protein pockets and ligand molecules. Later, we performed fine-tuning for tasks such as virtual screening, target fishing, and docking power. Experimental evaluations indicate that our approach consistently outperforms both traditional and state-of-the-art deep learning-based methods across multiple benchmarks.

## 2 Materials and methods

### 2.1 Related work

#### 2.1.1 Scoring function

Scoring functions play a crucial role in drug discovery. Traditional scoring functions primarily rely on physics-based approaches (An *et al.* 2015), including force field-based scoring functions, solvation models, and quantum mechanics methods. In parallel, empirical scoring functions (Zheng and Merz Jr 2011) estimate the binding affinity of complexes by aggregating key energetic contributions during the protein–ligand binding process (such as hydrogen bonds, hydrophobic effects, steric hindrance, etc.). Although scoring functions based on machine learning have surpassed traditional scoring functions in some scenarios (Cheng *et al.* 2012), their applicability often remains limited to specific cases, making sustained deployment in real-world workflows challenging. In contrast, our proposed DrugBLIP is designed for broad applicability across diverse scenarios, offering the potential to serve as a comprehensive replacement for traditional scoring functions.

#### 2.1.2 Multi-task learning

Multi-task learning (MTL) is a learning paradigm that effectively leverages both task-specific and shared information to address multiple related tasks simultaneously. Liu *et al.* (2019) proposed several architectures based on the shared-backbone idea, enabling the realization of multiple functions through a single network. Liu *et al.* (2019) further introduced specific task modules integrated within the shared architecture to enhance task performance. Li *et al.* (2022) proposed modal fusion strategies that combine vision and natural language representations, achieving competitive results on multiple vision–text tasks. Inspired by these works, we design DrugBLIP to use a unified backbone network capable of performing the diverse roles traditionally fulfilled by separate scoring functions.

### 2.2 Model architecture

**Figure 1:**
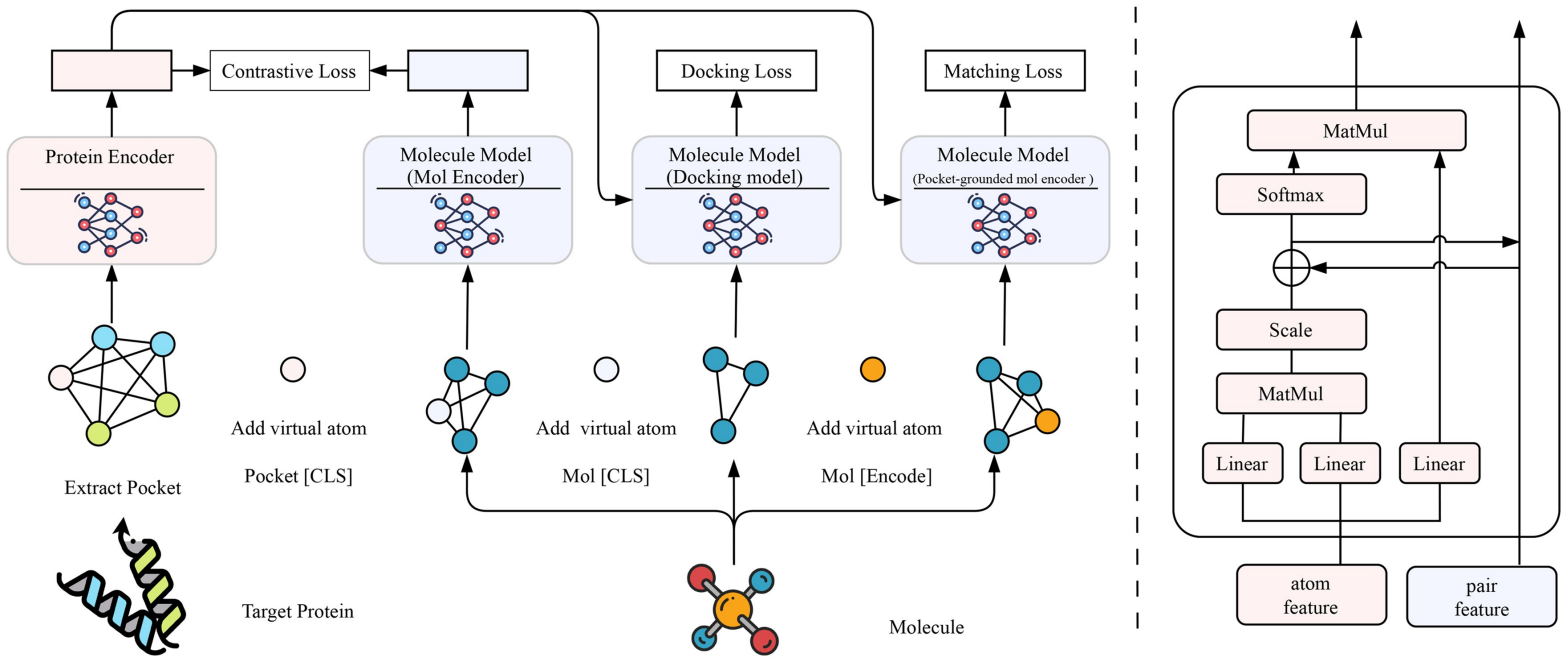
Overview of DrugBLIP. We propose a hybrid structure that can unify the representations of proteins and molecules for learning the interaction between proteins and molecules. It consists of three parts: (i) The Protein Pocket Encoder and Molecular Encoder are trained through a Contrastive loss to align the representations between proteins and molecules. (ii) The Docking Model models the relative position information between proteins and molecules by constructing a fully connected graph of proteins and molecules. While achieving docking, it outputs affinity ranking scores. (iii) The Protein Pocket-grounded mol Encoder is trained through matching loss to distinguish positive and negative protein pocket-molecule pairs.

As shown in Fig. 1, the overall flow of DrugBLIP consists of two primary components. The first is the **Protein Pocket Encoder**. Although the architecture supports full protein structures, in this study, we specifically focus on the binding pocket region to reduce noise and computational cost. The encoder takes the atomic graph of the protein pocket as input.

To ensure invariance to coordinate rotations and translations while capturing local chemistry, we adopt a dual-representation strategy maintaining both atom-level and pair-level features.

- **Atom Representation:** Each atom *i* is initialized with a feature vector $a_{i}^{0}$ derived solely from its atom type via an embedding layer, which is inherently invariant to SE(3) transformations.
- **Pair Representation:** Simultaneously, the pair representation $q_{ij}^{0}$ is initialized by combining Gaussian RBF-encoded Euclidean distances with bond type embeddings.

The RBF kernel maps squared Euclidean distances into a high-dimensional similarity measure:


(1) $$K(x_{i},x_{j})=\text{exp}\;\left(-\gamma \cdot ∥x_{i}-x_{j}{∥}^{2}\right),$$

Crucially, using this RBF encoding to initialize the pair representation anchors the geometric awareness of the model at the input stage, serving as an invariant 3D spatial positional encoding.

The backbone is based on a Transformer modified for 3D spatial data. To facilitate information exchange between 3D geometry and semantic features, the model performs iterative updates:

1. **Atom-to-pair communication:** The pair representation $q_{ij}$ is updated using the attention map from the self-attention mechanism. Formally, for layer *l*:
  (2) $$q_{ij}^{l+1}=q_{ij}^{l}+\left\{\frac{Q_{i}^{l,h}{\left(K_{j}^{l,h}\right)}^{T}}{\sqrt{d}}|h\in [1,H]\right\},$$
  where *H* is the number of heads and *d* is the hidden dimension.
2. **Pair-to-atom communication:** To leverage 3D information in atom updates, the pair representation acts as a bias term in the self-attention calculation:
  (3) $$\text{Attention}(Q_{i},K_{j},V_{j})=\text{softmax}\left(\frac{Q_{i}K_{j}^{T}}{\sqrt{d}}+q_{ij}^{l-1}\right)V_{j}.$$

This mechanism ensures that the attention weights are modulated by the geometric distance and bond information encoded in $q_{ij}$, rendering the internal representations invariant to global rotation and translation.

Finally, to predict 3D structural changes (docking poses), an SE(3)-equivariant head predicts delta positions (coordinate updates). It uses the SE(3)-invariant pair features and the equivariant vector difference $\left(x_{i}-x_{j}\right)$:


(4) $$\begin{array}{c} {\hat{x}}_{i}=x_{i}+\frac{1}{n}\sum_{j=1}^{n}\left(x_{i}-x_{j}\right)c_{ij},c_{ij}=\text{ReLU}\left((q_{ij}^{L}-q_{ij}^{0})U\right)W, \end{array}$$

where *U* and *W* are projection matrices. Note that $c_{ij}$ is derived from the difference in pair representations $\left(q_{ij}^{L}-q_{ij}^{0}\right)$, allowing the model to focus on relative structural refinement.

To train a unified model for understanding protein–molecule interactions, we adopt a multimodal hybrid approach including three modules. By encoding both atomic and 3D information, a virtual atom Pocket “[CLS]” is inserted into the Pocket Encoder. It is initialized with a learnable embedding vector, placed at the geometric center, and connected to all real atoms. The specific modules are:

1. **Mol encoder**: Adds a virtual atom Mol “[CLS]” located at the molecular center. Following the same initialization strategy as the Pocket “[CLS],” it is assigned a learnable embedding, placed at the geometric center, and connected to all molecular atoms.
2. **Docking model**: Processes the complete protein pocket–molecule pair and outputs docking scores for downstream screening. Feature representations from the respective encoders are fused through a global interaction stage, where the molecule is positioned relative to the protein pocket before scoring.
3. **Pocket-grounded mol encoder**: Introduces a virtual atom “[Encode]” at the molecular center. This token is initialized identically (using a learnable embedding at the geometric center) to encode cross-context information between the protein pocket and the molecule.

### 2.3 Pre-training objectives

Traditional force-field-based methods can simultaneously perform various functions for proteins and molecules, such as virtual screening and protein–ligand docking. Inspired by this and previous work (Li *et al.* 2022), we pre-train DrugBLIP with three objectives, each targeting a specific aspect of protein–molecule correlation. Two objectives focus on property understanding, and one on positional generation. Each protein pocket–molecule pair only requires a single forward pass through the computationally intensive protein encoder and three forward passes through the mixed model. These distinct functionalities are jointly optimized via the following loss functions.

#### 2.3.1 Protein pocket–molecule contrastive loss

Contrastive loss is used to align the feature spaces of the protein pocket encoder and the molecule model. Existing studies (Singh *et al.* 2023, Bhat *et al.* 2025) have also applied contrastive learning to molecular data; however, most rely on protein sequences or molecular fingerprints. In contrast, we perform structure-level alignment between proteins and small molecules. This encourages similar feature representations for positive pocket–molecule pairs and repels negative pairs.

We aim to learn a similarity function *s* such that paired pocket-molecule structures have higher similarity scores. Let $v_{\text{cls}}^{P}$ and $v_{\text{cls}}^{M}$ be the embeddings of the “[CLS]” tokens from the Protein and Mol encoders, respectively. We use linear transformations $g_{P}(\cdot )$ and $g_{M}(\cdot )$ to map these embeddings to normalized lower-dimensional representations. The similarity between a protein pocket *P* and a molecule *M* is defined as the dot product of their projected features:


(5) $$s(P,M)=g_{P}{\left(v_{\text{cls}}^{P}\right)}^{⊤}g_{M}(v_{\text{cls}}^{M}).$$

For a mini-batch of *N* pairs, we calculate the softmax-normalized pocket-to-molecule and molecule-to-pocket similarity. For the *i*th pair $\left(P_{i},M_{i}\right)$:


(6) $$\begin{array}{c} p_{i}^{p2m}(P_{i})=\frac{\;\text{exp}(s(P_{i},M_{i})/\tau )}{\sum_{k=1}^{N}\;\text{exp}\;(s(P_{i},M_{k})/\tau )},\\ p_{i}^{m2p}(M_{i})=\frac{\;\text{exp}(s(M_{i},P_{i})/\tau )}{\sum_{k=1}^{N}\;\text{exp}\;(s(M_{k},P_{i})/\tau )}, \end{array}$$

where τ is a learnable temperature parameter.

Let $y^{p2m}(P_{i})$ and $y^{m2p}(M_{i})$ denote the ground-truth one-hot similarity, where the positive pair $\left(P_{i},M_{i}\right)$ has a probability of 1 and negative pairs have 0. The contrastive loss is defined as the cross-entropy CE between the predicted probability p and the ground truth y:


(7) $$\begin{array}{c} L_{\text{pmc}}=\frac{1}{2}E_{(P,M)∼D}[\text{CE}(y^{p2m}(P),p^{p2m}(P))\\ +\text{CE}(y^{m2p}(M),p^{m2p}(M))]. \end{array}$$

#### 2.3.2 Protein pocket–molecule matching loss

This objective captures fine-grained compatibility between a protein pocket and a molecule by treating the task as binary classification. The output embedding of the virtual atom “[Encode],” which aggregates cross-modal information, is fed into a linear classifier to predict whether the pair is matched (positive) or unmatched (negative). To enhance discriminability, we adopt hard negative mining. Specifically, we select negative pairs that are incorrectly predicted with high similarity scores in the contrastive learning stage. The loss is defined as:


(8) $$L_{\text{pmm}}=E_{(P,M)∼D}\text{CE}(y^{\text{pmm}},p^{\text{pmm}}(P,M)),$$

where $y^{\text{pmm}}$ is the 2D one-hot ground-truth label, and $p^{\text{pmm}}$ is the predicted probability.

#### 2.3.3 Protein pocket–molecule docking loss

This objective enables the model to predict accurate binding positions by explicitly optimizing the geometry structure. The loss consists of two components: (i) **Intra-molecular Loss** for maintaining the valid local structure of the molecule, and (ii) **Inter-molecular Loss** for determining the precise binding pose relative to the pocket.

For the internal structure, we use the Huber loss on pairwise atomic distances to ensure robustness against structural outliers (e.g. flexible rotatable bonds).


(9) $$L_{\text{intra}}=\frac{1}{\left|P_{\mathrm{mol}}\right|}\sum_{(i,j)\in P_{\mathrm{mol}}}H_{\delta }(d_{ij}-d_{ij}^{*}),$$

where $P_{\mathrm{mol}}$ is the set of atom pairs within the molecule, $d_{ij}$ and $d_{ij}^{*}$ are the predicted and ground-truth distances, respectively. $H_{\delta }(\cdot )$ is the Huber function with threshold δ:


(10) $$H_{\delta }(a)=\{\begin{array}{c} \frac{1}{2}a^{2}\;\text{if}\;|a|\leq \delta ,\\ \delta (|a|-\frac{1}{2}\delta )\;\text{otherwise}. \end{array}$$

For the binding pose, we use the Mean Squared Error (MSE) loss on the coordinate deviations to encourage high positional fidelity of the ligand atoms relative to the protein pocket.


(11) $$L_{\text{inter}}=\frac{1}{N_{\mathrm{mol}}}\sum_{i=1}^{N_{\mathrm{mol}}}∥{\hat{x}}_{i}-x_{i}^{*}{∥}^{2},$$

where $N_{\mathrm{mol}}$ is the number of atoms in the molecule, ${\hat{x}}_{i}$ is the predicted coordinate, and $x_{i}^{*}$ is the ground-truth coordinate.

The overall pre-training objective combines these terms:


(12) $$L=L_{\text{pmc}}+L_{\text{pmm}}+L_{\text{intra}}+L_{\text{inter}}.$$

### 2.4 Model pretraining

#### 2.4.1 Pre-training data

We constructed the pre-training dataset from the PDBBind database (Wang *et al.* 2005) and the CrossDocked 2020 dataset (Francoeur *et al.* 2020), selecting protein pockets and ligand molecules whose binding site deviation was <1 Å. Motivated by Gao *et al.* (2024), we used the HomoAug strategy for data augmentation. Unlike direct noise injection, which can introduce instability or chemically implausible structures, HomoAug replaces the original protein with a homologous counterpart, thereby generating new valid complex structures. All samples used in downstream evaluation were removed to prevent data leakage. This process yielded a pre-training dataset of 217 146 protein pocket–small molecule pairs.

#### 2.4.2 Pre-training settings

Our model is implemented in PyTorch and pre-trained on sixteen NVIDIA V100 GPUs. For both the Protein encoder and Mol encoder, we initialize weights from UniMol models pre-trained on unpaired data (Zhou *et al.* 2023). Training details for this initialization stage are provided in Supplementary B.1, available as supplementary data at *Bioinformatics* online. During pre-training, the Protein and Mol encoders are optimized jointly using the curated dataset. We adopt the AdamW optimizer with a weight decay of 0.05. The learning rate follows a 1000-step linear warm-up followed by cosine annealing, with a peak and minimum learning rate of $1\times 10^{-4}$ and $5\times 10^{-6}$, respectively. Additional hyperparameters and settings are listed in Supplementary B, available as supplementary data at *Bioinformatics* online.

### 2.5 Model fine-tuning

All fine-tuning experiments use the pre-trained parameters from the first stage for initialization.

#### 2.5.1 Fine-tuning DrugBLIP for drug virtual screening

The objective of virtual screening is to identify active molecules from a small-molecule library that can effectively bind to a given target protein. For this task, and to ensure a fair comparison with prior studies, we use true positive protein–ligand complexes with accurate structures from the PDBBind 2019 dataset. As with pre-training, all samples overlapping with test sets are removed, yielding 65 989 protein–ligand pairs for fine-tuning.

**Figure 2:**
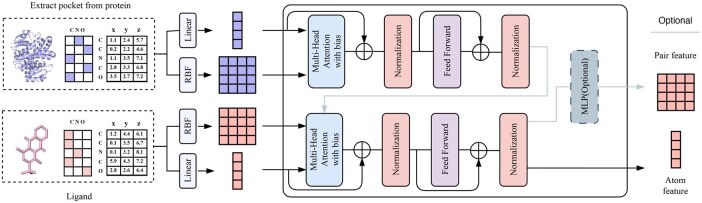
Model architecture. The model inputs the atom types and coordinates of Protein and Ligand. Obtain the atomic features through the atom types, calculate the distance through the coordinates, obtain the initial Pair features through RBF transformation. In the update process, it will pass through Multi-Head Attention. The Pair features are used as the bias of Attention. While updating the atom features, it is updated. According to different tasks, it can be selected whether to input the features of protein into the feature update process of Ligand. The updated Ligand features and protein features are used for downstream tasks.

As illustrated in Fig. 2, each candidate molecule is augmented with a virtual atom, denoted as Mol “[CLS],” placed at the molecular centroid, and passed to the Mol Encoder. Likewise, each protein pocket is augmented with a virtual atom, Protein “[CLS],” placed at the pocket centroid, before being fed into the Protein Encoder. The resulting molecular and protein feature embeddings are compared via a similarity function: higher similarity scores indicate a stronger likelihood of the molecule being an active compound, whereas lower scores suggest inactivity.

#### 2.5.2 Fine-tuning DrugBLIP for target fishing

Target fishing seeks to identify potential biological targets for known or candidate drug molecules. This approach deepens our understanding of target mechanisms of action, facilitates the investigation of side effects, helps address drug resistance, and ultimately improves therapeutic efficacy. It also enables the discovery of novel drug targets, broadens the applications of existing drugs, and supports drug repurposing, thereby offering more possibilities for disease treatment.

To ensure fair comparison with prior methods, we use the PDBbind General set v.2020, excluding complexes that overlap with the test set. Data preprocessing includes adding missing atoms to both proteins and ligands, manually correcting file-loading errors, and filtering pockets containing fewer than 5 residues within 5 Å of the ligand. We further remove complexes from PDBbind whose protein sequence identity to test proteins exceeds 40% [measured with MMseqs2 (Steinegger and Söding 2017)] or whose ligand fingerprint similarity to test ligands exceeds 80% [computed with RDKit (Landrum *et al.* 2013)].

As illustrated in Fig. 2, the molecule is augmented with a virtual atom labeled “[Encode]” placed at the molecular centroid. The candidate protein pocket is processed by the Protein Encoder, and its features are combined with those of the molecule containing the virtual atom before being passed to the Molecular Encoder. The “[Encode]” token facilitates feature alignment between the two modalities. The model outputs a matching score between the protein target and the molecule, along with a ranking that prioritizes target proteins most likely to bind effectively.

#### 2.5.3 Fine-tuning DrugBLIP for protein–molecule docking and ranking

Protein–molecule docking aims to predict both the ligand binding pose and the conformation of the resulting complex. This requires considering conformational changes in both partners as well as their relative positioning. Accurately modeling these interactions is critical for elucidating biological processes and for rational drug design.

Following the protocol of Zhou *et al.* (2023), we construct the docking training set from the PDBbind General set v.2020, a widely used benchmark comprising diverse protein–ligand complexes. Prior to training, we filter out erroneous or incomplete entries, retaining only complexes whose binding pockets contain at least five residues within 5 Å of the ligand. This yields 165 630 protein–ligand pairs.

As shown in Fig. 2, after protein features are extracted by the Protein Encoder, they are concatenated with molecule features within the Molecular Encoder equipped with a pair-wise MLP. Within this encoder, cross-modal interactions update the pair representations to capture fine-grained positional relationships between protein and ligand atoms. The updated features are projected into a distance space via the MLP, where intramolecular distances refine ligand conformation and intermolecular distances localize the ligand within the binding site.

## 3 Results

In this section, we conduct experiments on three functions commonly used in traditional scoring functions, including virtual screening, Protein–Ligand docking, and targetfishing to evaluate whether DrugBLIP can effectively learn the interaction between proteins and molecules. We conducted ablation experiments on different training strategies. The results can be found in the Supplementary C.1, available as supplementary data at *Bioinformatics* online.

### 3.1 Virtual screening

For virtual screening, we used DUD-E (Mysinger *et al.* 2012) as our test benchmark, which contains 102 proteins and their corresponding 22 886 bioactive molecules. We evaluated performance using common indicators in virtual screening, including AUROC, Boltzmann-enhanced Discrimination of ROC (BEDROC), and Enrichment Factor (EF). BEDROC incorporates exponential weights and assigns higher weights to top-ranked molecules.

In order to effectively evaluate the generalization of DrugBLIP, we adopted a zero-shot evaluation setting, meaning that no fine-tuning was performed on DUD-E. We compared our approach with various methods, including traditional techniques such as Δ-VinaRF and RFscore, as well as deep learning-based methods like TANKBind and EquiScore. The experimental results are shown in Table 1.

**Table 1:** Performance comparison between DrugBLIP and different baseline approaches on DUD-E datasets.^a^

Methods		AUROC		BEDROC		EF					
						0.5%		1%		5%	
		Mean	Median	Mean	Median	Mean	Median	Mean	Median	Mean	Median
Scoring Functions	pafnucy	0.6313	0.6389	0.1645	0.1450	4.24	2.64	3.86	2.59	2.77	2.41
	RFscorev1	0.6105	0.6295	0.1158	0.0540	3.11	1.54	2.76	1.50	2.24	1.69
	RFscorev2	0.6484	0.6602	0.1536	0.1180	6.19	4.05	5.56	4.14	3.47	3.29
	RFscorev3	0.6372	0.6447	0.1573	0.1084	6.68	3.73	5.78	1.90	2.41	3.08
	RFscorev4	0.6525	0.6546	0.1242	0.1044	4.90	3.07	4.52	3.27	2.98	2.76
	PLECRF	0.6370	0.6447	0.1819	0.1069	8.47	8.47	7.09	3.26	3.66	2.67
	Δ− VinaRF	0.6967	0.6954	0.2134	0.1489	9.52	5.56	8.00	5.52	4.38	3.66
	Glide SP	0.7670	0.7833	0.4073	0.4062	19.39	19.19	16.18	15.99	7.23	7.20
Deep Learning	OnionNet	0.5971	0.6065	0.0855	0.0855	2.84	1.77	2.84	1.77	2.20	2.09
	NNscore	0.6826	0.6796	0.1223	0.0963	4.16	2.75	4.02	3.18	3.12	2.53
	DeepDock	0.6503	0.6475	0.2180	0.1089	11.01	3.95	8.79	3.95	4.07	2.84
	3D-GNN	0.6620	0.6682	0.3273	0.2761	15.85	13.51	13.08	13.51	5.75	5.21
	KDeep	0.7083	0.7195	0.2278	0.2249	12.33	10.09	10.39	10.09	5.39	4.86
	RTMScore	0.7329	0.7460	0.4506	0.4719	23.19	25.63	19.04	25.63	7.27	7.20
	PIGNet	0.7556	0.7705	0.3620	0.3362	16.73	14.34	14.19	14.34	6.78	6.12
	EquiScore	0.7757	0.7893	0.4323	0.4260	20.94	22.10	17.67	22.10	7.82	7.46
	TANKBind	0.7779	0.8140	0.3303	0.2921	14.07	13.85	12.62	13.85	6.82	6.27
	DrugCLIP	0.8069	0.8571	0.5002	0.5051	38.94	42.84	31.97	31.51	10.65	11.06
Ours	DrugBLIP	**0.8217**	**0.8632**	**0.5743**	**0.5623**	**44.01**	**43.58**	**37.02**	**36.05**	**11.80**	**12.23**

a Bold indicate state-of-the-art.

On the DUD-E dataset, DrugBLIP achieved the best performance in terms of AUROC, scoring 0.8217, surpassing all baseline methods, including the latest deep learning approaches DrugCLIP (Gao *et al.* 2024) and EquiScore (Cao *et al.* 2024). Traditional methods often suffer from high false positive or false negative rates due to their limited ability to capture the complex interactions between proteins and ligands. Scoring functions (Wójcikowski *et al.* 2017) rely on force-field interactions and cannot flexibly adapt to diverse docking scenarios, and in practice are generally weaker than deep learning-based methods. Glide SP performs best among various scoring functions and even surpasses many deep learning methods; however, due to methodological limitations, it is very slow to run, though it exhibits higher stability across indicators and can replace deep learning methods in some scenarios. For deep learning methods, the use of large-scale training data and algorithmic advances can improve performance to some extent, but most prior methods are trained on a single task, limiting their ability to fully capture protein–ligand interactions.

DrugBLIP, by leveraging advanced protein–molecule interaction mechanisms learned from multi-task training and graph transformer-based models, shows remarkable performance. Compared with the previous best scoring function, DrugBLIP achieves a 41% improvement in BEDROC. For the more challenging enrichment factor metric, EF 0.5% increases by 127%, EF 1% by 128.8%, and EF 5% by 63.2%. Compared with the best deep learning method, it achieves a 14.8% improvement in BEDROC, with EF 0.5% increased by 13.0%, EF 1% by 15.8%, and EF 5% by 10.8%. This significant improvement demonstrates the effectiveness of DrugBLIP.

To further verify that the above improvements are not due to potential data leakage between training and test sets, we conducted a protein-level deduplication experiment on DUD-E. Specifically, we removed from the training set any complex whose protein shared the same UniProt ID as a protein in the DUD-E test set, ensuring that all proteins in the test set were unseen during both pre-training and fine-tuning. The detailed procedure and dataset statistics after deduplication are provided in the Supplementary Materials, available as supplementary data at *Bioinformatics* online (Extended Data Table 1). Under this stricter evaluation protocol, DrugBLIP still achieved strong results (AUROC 0.7311, BEDROC 0.3432, EF 0.5% 21.37, EF 1% 18.75, EF 5% 8.06), outperforming all traditional scoring functions and deep learning baselines. This confirms that DrugBLIP maintains a clear advantage even when evaluated on entirely unseen protein targets, highlighting its robust generalization capability.

### 3.2 Docking power

We evaluated the docking power of DrugBLIP, particularly its ability to distinguish native binding poses from those generated by established docking software packages. Our assessment primarily focused on the benchmark datasets CASF-2013 and CASF-2016. A pose is considered native if its root mean square deviation (RMSD) from the true binding pose is <2 Å. During docking, the molecular encoder facilitates the movement of the molecule to the correct docking position based on the interactions between the molecule and the protein, and it outputs a docking score. The CASF-2013 dataset contains 195 protein–ligand pairs along with multiple decoys, while the CASF-2016 dataset comprises 285 protein–ligand pairs with multiple decoys. We compare DrugBLIP against a range of widely used scoring functions to provide a comprehensive performance benchmark.

As shown in Table 2, DrugBLIP exhibited outstanding performance on CASF-2013, achieving a top-1 success rate of 90.27%, surpassing methods such as AutoDock Vina. Additionally, it outperformed previous methods in top-2 and top-3 success rates, reaching 92.82% and 94.36%, respectively. On CASF-2016, DrugBLIP achieved a top-1 success rate of 91.23%, exceeding all prior methods. While its top-2 and top-3 success rates are marginally lower than those of a few earlier approaches, the top-1 metric is often the most relevant for real-world applications, as it reflects the ability to identify the correct pose in the first prediction. This underscores the diversity and potential of the proposed method in enhancing docking accuracy, providing a comprehensive and innovative solution to docking challenges.

**Table 2:** Performance on CASF 2016 and CASF 2013 benchmark.^a^

Method	CASF 2016			CASF 2013		
	Top1	Top2	Top3	Top1	Top2	Top3
X-Score	65.3	77.9	83.5	61.0	71.3	76.4
ChemScore	80.4	86.0	90.9	61.0	84.1	88.7
ASP	81.1	88.4	93.0	73.3	82.1	87.7
GoldScore	75.1	86.3	90.5	72.3	84.1	88.7
dSAS	30.2	44.6	51.6	26.2	37.9	58.7
LigScore2	85.6	93.3	96.5	78.5	84.6	87.2
GlideScore-SP	87.7	91.9	93.7	79.0	86.7	88.7
ChemPLP	86.0	93.7	96.1	81.0	86.7	89.7
AutodockVina	90.2	**95.8**	**97.2**	85.6	90.8	92.8
DrugBLIP	**91.2**	94.7	96.1	**90.3**	**92.8**	**94.4**

a Here, top1, 2, and 3 are defined as the proportion of top1, top2, and top3 with rmsd <2 Å. This is defined as the success rate with the unit of %. Bold indicates the best performance, and underline indicates the second-best.

We sought to understand why DrugBLIP achieves such high docking power. To investigate this, we compared the positions of the ligand before and after docking. It is evident that when the initial position of the ligand is far from the correct binding site, the movement of the molecule is substantial; conversely, when the ligand starts from a position close to the correct binding site, the movement is minimal. This indicates that DrugBLIP can effectively discern whether a given initial position is correct when faced with various starting configurations. Representative case studies illustrating these behaviors are provided in Supplementary C.2, available as supplementary data at *Bioinformatics* online. If the initial position deviates significantly from the correct binding site, the model docks the molecule to the correct position, resulting in greater movement. On the other hand, if the initial position is already close to the correct binding site, only minimal adjustments are needed. DrugBLIP learns this behavior through prior docking experiences, which contributes to its robust docking capabilities. Furthermore, we compared the efficiency of DrugBLIP with that of common docking tools, as reported in Supplementary C.3, available as supplementary data at *Bioinformatics* online, showing that DrugBLIP achieves competitive or superior runtime performance while maintaining high accuracy.

### 3.3 Target fishing

To further validate the interactions between protein pockets and molecules facilitated by DrugBLIP, we evaluated its performance on the *target fishing* task. The core idea of target fishing is to identify potential biological targets from known or candidate drug molecules through computational or experimental methods (Chen *et al.* 2020). This approach aids in understanding the mechanisms of target action, addressing issues of resistance and side effects, enhancing the efficacy of drug treatments, and expanding the applications of drugs. Additionally, it enables drug repurposing, providing more possibilities for disease treatment.

Table 3 shows the performance of various methods on the CASF-2016 benchmark. From the perspective of traditional methods, X-Score, LigScore2, AutoDock Vina, etc. exhibit relatively low performance on Top-1, Top-5, and Top-10 indicators. For example, the Top-1 success rate of X-Score is only 7.00%, indicating that these methods have limited ability to accurately identify the most likely protein targets. Most are based on traditional docking algorithms or empirical scoring functions, making it difficult to fully capture the complex interaction patterns between small molecules and proteins, which leads to higher false positive or false negative rates in target fishing.

**Table 3:** Performance on CASF 2016 benchmark.^a^

Method	Top 1	Top 5	Top 10
X-Score (Zhang *et al.* 2009)	7.00	13.30	18.20
LigScore2 (Krammer *et al.* 2005)	11.20	17.50	29.50
AutodockVina (Trott and Olson 2010)	13.70	22.80	31.20
GlideScore-XP (Friesner *et al.* 2006)	14.40	23.50	34.70
VinaRF20 (Wang *et al.* 2021)	15.10	24.90	31.60
DrugScoreCSD (Velec *et al.* 2005)	15.40	23.90	33.00
GlideScore-SP (Friesner *et al.* 2006)	16.50	27.00	37.50
ChemPLP (Liebeschuetz *et al.* 2012)	17.50	29.10	41.10
DrugCLIP (Gao *et al.* 2024)	36.12	78.71	86.31
DrugBLIP (Ours)	**41.83**	**86.69**	**93.16**

a Here, top1, 5, and 10 are defined as the proportion of top1, top5, and top10. This is defined as the success rate with the unit of %. Bold indicates the best performance, and underline indicates the second-best.

With the development of technology, some improved methods such as GlideScore-XP, VinaRF20, and DrugScoreCSD have achieved better performance. The Top-1 success rate of GlideScore-XP reaches 14.40%, which is a certain improvement compared to traditional methods, yet it still falls short of the requirements for high-precision target fishing. These methods benefit from advances in molecular conformation prediction and scoring function optimization, but due to the limitations of their underlying principles, there remains substantial room for improvement when dealing with complex protein–small molecule interactions.

Among deep learning-based methods, DrugCLIP shows significant advantages, with Top-1, Top-5, and Top-10 success rates of 36.12%, 78.71%, and 86.31%, respectively, leading among existing methods and demonstrating the potential of deep learning in target fishing tasks. However, our proposed DrugBLIP achieves even higher accuracy: a Top-1 success rate of 41.83%, Top-5 of 86.69%, and Top-10 of 93.16%. By leveraging a multi-task trained graph Transformer architecture, DrugBLIP can more effectively learn deep-level features of protein–small molecule interactions and accurately capture key interaction patterns, thereby delivering superior performance in the target fishing task.

## 4 Conclusion

In this paper, we propose DrugBLIP, a machine learning model based on a SE(3) graph transformer designed to learn the interactions between proteins and molecules. As the first model to unify contrastive learning, matching learning, and docking tasks, it enables the model to understand the protein–molecule interaction process more comprehensively. Through multi-task learning, it effectively captures the protein–molecule interaction mechanism and achieves excellent performance on tasks such as virtual screening, docking, and target prediction. DrugBLIP is also expected to accelerate the drug development process, reduce research and development costs, and increase the success rate of drug development. In the future, we will continue to expand the functions of the model to enable it to support more types of biological macromolecules and design capabilities.

## Supplementary Material

btag069_Supplementary_Data

## Data Availability

The data underlying this article are available in Github at https://github.com/Wolkenwandler/DrugBLIP.
