# Supplementary material for: DrugBLIP: exploring the protein–molecule interaction mechanisms with a multi-task learning graph transformer
Source: Bioinformatics. 2026 Apr 10;42(4):btag069. doi: 10.1093/bioinformatics/btag069 (PMC13080933; doi:10.1093/bioinformatics/btag069)
Supplement: btag069_Supplementary_Data [file btag069_supplementary_data.pdf]

A. Proof of Equivariance

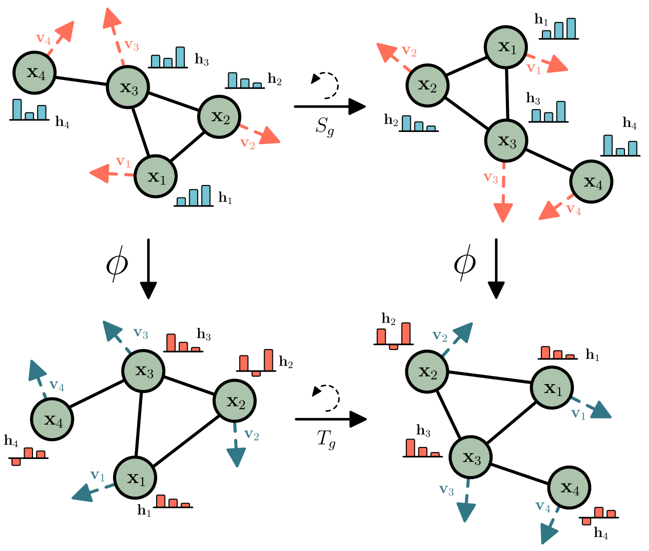

Figure 1: Example of Rotation Equivariance on a Graph with a Graph Neural Network (Referenced from Satorras et al. (2021))

In the realm of 3D spatial modeling, ensuring the equivariance property of a model is crucial for its effectiveness in handling 3D geometric information. Here, we present a detailed proof of the equivariance of the SE(3)-equivariant head introduced for predicting 3D positions. An SE(3) transformation in 3D space combines a rotation $R \in SO(3)$ (a 3×3 orthogonal matrix with $\det(R) = 1$) and a translation $t \in \mathbb{R}^3$. For an input vector $x_i \in \mathbb{R}^3$ (representing the coordinate of the $i-$th atom), the transformation is defined as $x_i' = Rx_i + t$.

The key input for the SE(3)-equivariant head in calculating the position update is $x_i - x_j$. Let's consider the transformation of this input under SE(3).

$$\begin{aligned}(x_i - x_j)' &= (Rx_i + t) - (Rx_j + t) \\ &= Rx_i + t - Rx_j - t \\ &= R(x_i - x_j)\end{aligned} \tag{1}$$

This shows that $x_i - x_j$ is equivariant under SE(3) transformation. The pair representation q_{ij} is designed to be invariant under global rotation and translation. That is, for any SE(3) transformation, q_{ij} remains unchanged. So, $(q_{ij}^L - q_{ij}^0)$ also maintains its value under SE(3) transformation.

Since $(q_{ij}^L - q_{ij}^0)$ is invariant under SE(3) transformation, and matrix multiplications with $U \in \mathbb{R}^{H \times H}$ and $W \in \mathbb{R}^{H \times 1}$ and the ReLU function ($\mathrm{ReLU}(y) = \max(0, y)$) do not disrupt this invariance property, c_{ij} is invariant under SE(3) transformation. The formula for calculating the output coordinate $\hat{x}i$ is

$$\hat{x}i = x_i + \sum_{j=1}^{n} \frac{(x_i - x_j)c_{ij}}{n}. \tag{2}$$

After an SE(3) transformation, the new $\hat{x}i'$ is:

$$\begin{aligned}\hat{x}i' &= (Rx_i + t) + \sum_{j=1}^{n} \frac{(R(x_i - x_j))c_{ij}}{n} \\ &= Rx_i + t + \sum_{j=1}^{n} \frac{R((x_i - x_j)c_{ij})}{n} \\ &= R\left(x_i + \sum_{j=1}^{n} \frac{(x_i - x_j)c_{ij}}{n}\right) + t \\ &= R\hat{x}_i + t\end{aligned} \tag{3}$$

This mathematical derivationdemonstrates that when the input x_i undergoes an SE(3) transformation, the output $\hat{x}_i$ of the SE(3)-equivariant head also undergoes the corresponding SE(3) transformation.

B. Implementation details

This chapter introduces some details of training. The entire large training process can be divided into three stages. In the first stage, unpaired data is used to pre-train two Encoders. In the second stage, paired data is used for multi-task pre-training. In the third stage, fine-tuning of each task is performed.

B.1. Encoder Pretraining

In the early stage of training, the pretraining strategy follows UniMol (Zhou et al., 2023), and the Encoder for proteins and the Encoder for molecules are pretrained separately. This part of the training uses unpaired data. The pretraining dataset used for molecules contains 19 million molecules. In the process of generating 3D conformations, ETKGD (Riniker and Landrum, 2015) combined with the Merck molecular force field (Halgren, 1996) is used for optimization in RDKit (Landrum et al., 2013). Eleven conformations are randomly generated for each molecule, totaling 209 million conformations. The pretraining dataset for candidate protein pockets is derived from the protein database (Berman et al., 2000), which contains 180,000 protein structure data. Firstly, the raw data is preprocessed by adding missing side chains and hydrogen atoms. Then, Fpocket (Le Guilloux et al., 2009) is used to detect candidate binding pockets of proteins. After screening the original pockets according to the number of residues (10 - 25) in contact with the pocket and including the water molecules inside the pocket, a pretraining dataset containing 3,291,739 candidate pockets is obtained.

During the training process, a self-supervised task of 3D position recovery is used. Given the disrupted input positions, the correct 3D positions are recovered. In practice, during training, noise with a random noise range of $r = 1\AA$ is added to the real positions. For the disrupted input coordinates, the model needs to predict the correct Euclidean distances between disrupted atom pairs and perform coordinate prediction. Based on the SE(3)-equivariant coordinate head, the model needs to predict the correct coordinates of the disrupted atoms. Finally, the atomic types of the disrupted atoms are masked, and a head is used to predict the correct atomic types. To facilitate fine-tuning, we use a special atom [CLS] whose coordinates are at the center of all atoms to represent the entire molecule/pocket. More training details can be referred to Unimol (Zhou et al., 2023).

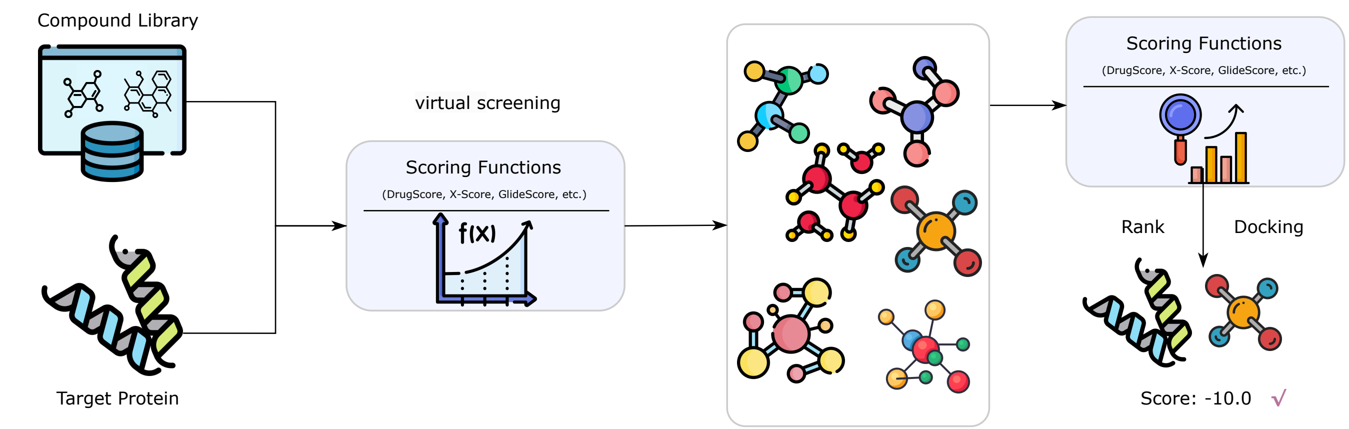

Figure 2: Overview of computer-aided drug design. After determining the target pocket, the way to obtain the drug corresponding to the target pocket is to obtain candidate drugs through virtual screening by scoring functions. After obtaining candidate drugs, they can be further sorted and scored by scoring functions for initial screening, and then drug experiments can be conducted.

B.2. Multi-task pre-training

We separately load the pretrained but previously unpaired weights for the Encoder of protein pockets and the Encoder of molecules. We train the model using the AdamW optimizer (Kingma and Ba, 2015), with a weight decay of 0.05. The learning rate scheduler uses a combination of 1000-step linear warm-up and cosine annealing. The peak learning rate and minimum learning rate are 1e-4 and 5e-6 respectively. The batch size is set to 32. he training objective is as follows: Equation **??**. We rewrite it as follows:

$$\mathcal{L} = \mathcal{L}_{\text{pmc}} + \mathcal{L}_{\text{pmm}} + \mathcal{L}_{\text{dist}} + \mathcal{L}_{\text{cross\_dist}}. \tag{4}$$

During the training process, 16 V100 cards are used. The total training time is two days.

B.3. Model Fine-tuning

Fine-tuning DrugBLIP for drug virtual screening. The objective of virtual screening is to identify active molecules from a small molecule database that can effectively bind to the target protein with biological activity. For virtual screening tasks, to ensure fair comparison with previous methods, we used the true positive protein-ligand complexes with accurate structures from PDBBind 2019 as the fine-tuning dataset, totaling 65,989 protein-ligand pairs.

Candidate small molecules are augmented with a virtual atom, denoted as Mol "[CLS]", positioned at the origin, and subsequently input into the Mol Encoder. Similarly, proteins are supplemented with a virtual atom, labeled Protein "[CLS]", also located at the origin, before being input into the Protein Encoder. The features extracted from the molecules and proteins by the model are then used to calculate similarity. A high similarity indicates that the molecule is a potential active compound, while a low similarity suggests that it does not meet the screening criteria.

We use the weights from multi-task pre-training as the initial weights for the task. On the V100 GPU, we use the same optimizer and learning rate scheduler configuration as in the first stage to fine-tune the checkpoints from the first stage and evaluate on DUD-E. This training process takes 10 hours.

Fine-tuning DrugBLIP for target fishing. The core concept of target fishing is to identify potential biological targets based on known or candidate drug molecules. This approach enhances our understanding of the mechanisms of action of these targets, aids in the investigation of side effects, addresses issues of drug resistance, and improves therapeutic efficacy. Furthermore, it facilitates the exploration of novel drug targets, broadens the applications of existing drugs, and enables drug repurposing, thereby offering more possibilities for disease treatment. To ensure fair comparison with previous methods, we used the true positive protein-ligand complexes with accurate structures from PDBBind 2019 as the fine-tuning dataset, totaling 65,989 protein-ligand pairs.

We use the weights from multi-task pre-training as the initial weights for the task. On the V100 GPU, we use the same optimizer and learning rate scheduler configuration as in the first stage to fine-tune the checkpoints from the first stage and evaluate on CASF-2013 and CASF-2016. This training process takes 8 hours.

Fine-tuning DrugBLIP for protein and molecular docking. The docking of proteins and molecules aims to predict the binding position of the protein and the structure of the complex formed with the molecular ligand. This process involves not only considering the conformational changes of both the protein and the molecule but also taking into account the relative positioning between them. Accurate prediction of these interactions is crucial for understanding biological processes and designing effective drugs.

We use the weights from multi-task pre-training as the initial weights for the task. On the V100 GPU, we use the same optimizer and learning rate scheduler configuration as in the first stage to fine-tune the checkpoints from the first stage and evaluate on CASF-2016. This training process takes 12 hours.

B.4. Evaluation Metrics

For virtual screening tasks, there are some metrics that are not very common in these tasks. We will introduce them below.

AUROC is the area under the receiver operating characteristic curve, is an important indicator for measuring the performance of binary classification models. In binary classification problems, the model predicts samples and gives the probability of belonging to the positive class. By setting different classification thresholds,

Table 1. Ablation experiment results on the impact of different training strategies on results

Ablation Settings	AUROC	BEDROC	EF		
			0.5%	1%	5%
DrugBLIP	0.8217	0.5743	44.01	37.02	11.80
(w/o) Multi-Task Training	0.8050	0.3552	24.70	21.25	9.05
(w/o) Train Pocket Encoder	0.7305	0.2854	21.05	17.06	7.05
(w/o) Pocket Feature	0.6952	0.1285	6.69	5.96	4.45
(w/o) Pocket Feature & Train Interaction	0.5357	0.0336	1.84	1.60	1.30
(w/o) Finetune Mol Encoder	0.8036	0.4399	33.06	27.86	9.67
(w/o) Train Interaction	0.3818	0.0136	0.60	0.77	0.60

Table 2. *Performance comparison between DrugBLIP and different baseline approaches on unseen DUD-E datasets. Bold indicate state-of-the-art.*

Methods		AUROC	BEDROC	EF		
				0.5%	1%	5%
Scoring Functions	pafnucy	0.6296	0.1409	3.46	3.07	2.23
	RFscorev1	0.5617	0.0518	1.74	1.49	1.56
	RFscorev2	0.6062	0.1033	4.44	3.81	2.37
	RFscorev3	0.5933	0.1221	5.60	4.78	2.77
	RFscorev4	0.6078	0.0887	3.39	3.11	2.28
	PLECRF	0.5644	0.0867	3.28	3.44	2.26
	Δ−VinaRF	0.6665	0.1778	7.05	5.71	3.49
	Glide SP	**0.7422**	**0.4041**	17.60	14.02	6.65
Deep Learning	OnionNet	0.5335	0.0489	1.24	1.24	1.26
	NNscore	0.6761	0.1580	3.96	3.86	3.29
	DeepDock	0.6376	0.1476	5.14	4.85	3.73
	3D-GNN	0.5866	0.1507	4.91	4.32	3.02
	KDeep	0.6606	0.2008	9.25	7.79	3.91
	RTMScore	0.6584	0.2953	10.60	9.32	5.31
	PIGNet	0.6736	0.1994	7.90	6.72	3.88
	EquiScore	0.7123	0.3139	12.93	10.36	5.59
	TANKBind	0.5833	0.0757	2.17	2.16	1.88
	DrugCLIP	0.6500	0.1688	10.18	9.70	4.09
Ours	DrugBLIP	0.7311	0.3432	**21.37**	**18.75**	**8.06**

different true positive rates (TPR) and false positive rates (FPR) can be obtained. The curve drawn with FPR as the horizontal axis and TPR as the vertical axis is the ROC curve. TPR = TP / (TP + FN), where TP is the number of true positive cases and FN is the number of false negative cases; FPR = FP / (FP + TN), FP is the number of false positive cases, and TN is the number of true negative cases. AUROC is the area covered under the ROC curve. Its value range is between 0 and 1. The closer the value is to 1, the better the model performance is, which means that the model can effectively distinguish positive and negative samples; if it is close to 0.5, it means that the model's distinguishing ability is equivalent to random guessing. Since the ROC curve is not a regular figure in practice, numerical calculation methods such as the trapezoidal rule are often used to approximately calculate AUROC. The formula is

$$\mathrm{AUROC} \approx \sum_{i=1}^{n-1} \frac{(\mathrm{FPR}_{i+1} - \mathrm{FPR}_i)(\mathrm{TPR}_{i+1} + \mathrm{TPR}_i)}{2}, \quad (5)$$

where n is the number of points on the ROC curve, and FPR_i and TPR_i are the false positive rate and true positive rate corresponding to the i-th point respectively.

BEDROC incorporates exponential weights that assign greater importance to early rankings. In the context of virtual screening, the commonly used variant is BEDROC_{85}, where the top 2% of ranked candidates contribute to 80% of the BEDROC score (Gao et al., 2024). The formal definition is:

$$\mathrm{BEDROC}_\alpha = \frac{\sum_{i=1}^{\mathrm{NTB}_t} e^{-\alpha r_i/N}}{R_\alpha(\frac{1-e^{-\alpha}}{e^{\alpha/N}-1})} \times \frac{R_\alpha \sinh(\alpha/2)}{\cosh(\alpha/2) - \cosh(\alpha/2 - \alpha R_\alpha)} + \frac{1}{1 - e^{\alpha(1-R_\alpha)}}. \quad (6)$$

Enrichment Factor (EF), is an index used in fields such as chemical screening and drug research and development to measure the enrichment effect of screening methods or experimental processes on target compounds. It reflects the improvement in the

enrichment degree of target compounds in a selected portion of samples through specific screening methods compared to random selection. Its calculation formula is

$$\mathrm{EF}_\alpha = \frac{\mathrm{NTB}_\alpha}{\mathrm{NTB}_t \times \alpha}, \quad (7)$$

where NTB_α is the number of true binders in the top $\alpha\%$ and NTB_t is the total number of binders in the entire screening pool.

C. Other Results

In this chapter, we conducted an ablation study and reported a case study.

C.1. Ablation Study

To deeply explore the impact of each key component and training strategy of the DrugBLIP model on its performance, we conducted a detailed ablation experiment. The experimental results are shown in Table **??**. Among multiple indicators for evaluating model performance, AUROC, BEDROC, and EF under different screening ratios are important measurement criteria. The experimental results show that the complete DrugBLIP model performs the best. Its AUROC reaches 0.8217, and BEDROC is 0.5743. In terms of the EF indicator, the values under screening ratios of 0.5%, 1%, and 5% are 44.01, 37.02, and 11.80 respectively.

When multi-task training is removed (w/o Multi-Task Training, i.e., excluding all auxiliary losses and retaining only the virtual screening objective), all performance indicators of the model show a significant decline. AUROC drops to 0.8050, BEDROC drops significantly to 0.3552, and the EF indicator also decreases significantly. This fully shows that multi-task training plays a key role in enabling the DrugBLIP model to learn rich features and improve predictive ability.

If the pocket encoder is not trained (w/o Train Pocket Encoder, freezing the parameters of the pocket encoder during fine-tuning), the model performance declines more significantly. AUROC drops to 0.7305, BEDROC is 0.2854, and the EF indicator also decreases significantly. This highlights the importance of the pocket encoder in capturing protein pocket features.

The setting (w/o Pocket Feature) corresponds to the ligand-only fine-tuning baseline: during fine-tuning, only ligand features are used for scoring, with protein features masked out; at test time, both inputs are provided but the protein branch does not contribute to the score. This yields AUROC 0.6952 and BEDROC 0.1285, indicating that removing protein information substantially weakens the model. The variant (w/o Pocket Feature & Train Interaction) further disables fine-tuning on the virtual screening task and still uses only ligand features, resulting in AUROC 0.5357 and BEDROC 0.0336, which confirms that both protein information and interaction modeling are critical.

When the molecular encoder is not fine-tuned (w/o Finetune Mol Encoder, freezing the molecular encoder during fine-tuning), the model performance also declines. AUROC is 0.8036, BEDROC is 0.4399, and the EF indicator is also affected to a certain extent. This shows that fine-tuning the molecular encoder helps the model better learn molecular features and thereby improve overall performance.

Finally, (w/o Train Interaction) disables fine-tuning of the interaction alignment between pocket and ligand representations, causing the most severe degradation (AUROC 0.3818, BEDROC 0.0136), underscoring that learning cross-modal interaction representations is essential. We note that a protein-only baseline is degenerate under our per-target virtual screening protocol: each target has only one protein structure, so such a model would assign identical scores to all ligands of the same target, yielding random-level performance by construction.

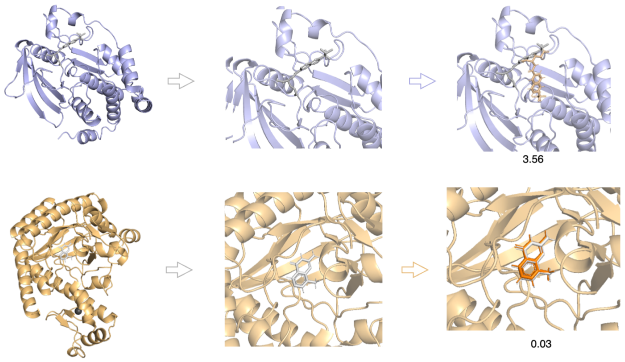

Figure 3: Two examples of the docking process. The change from the initial position to the final position. For ligands with a large or small initial position deviation, DrugBLIP can effectively move to the accurate position.

C.2. Case Study

The Figure 3 shows the excellent ability of DrugBLIP in handling ligand molecule positioning. The upper row shows the situation of ligand molecules with a large initial position deviation. From left to right, initially, the ligand molecule is in a relatively distant position in the protein structure. With the action of DrugBLIP, the ligand molecule gradually moves towards the key area of the protein and finally reaches an effective position. The output affinity-related value is 3.56, indicating that DrugBLIP can effectively guide ligand molecules with a large initial position deviation to move to a suitable position and achieve a reasonable combination with the protein. The lower row presents the situation of ligand molecules with a small initial position deviation. Also from left to right, at the beginning, the ligand molecule is close to a certain part of the protein. After the action of DrugBLIP, it is further accurately moved to an effective position. The corresponding value is 0.03, indicating that even for ligand molecules with a small initial deviation, DrugBLIP can further optimize its position to reach the best binding state. The above shows that when handling ligand molecules with different initial deviation states, DrugBLIP has the ability to move them to an effective position. This is of great significance in the research of protein-ligand interaction and drug design fields, proving the effectiveness and universality of DrugBLIP in optimizing ligand-protein binding.

C.3. Efficiency Compare

We compared DrugBLIP with common docking tools in terms of efficiency. DrugBLIP runs on a single V100 GPU in combination with pose prediction. For each target, DrugBLIP is executed with 10 different initial conformations and the total time consumption is recorded. In addition to traditional docking programs, we also compared against the recently proposed Boltz-2 methodpassaro2025boltz under the same hardware conditions. The

results show that DrugBLIP achieves a speed hundreds of times faster than conventional docking tools, and remains substantially faster than Boltz-2 while maintaining competitive accuracy. We compared DrugBLIP with common docking tools in terms of efficiency. DrugBLIP runs on a single V100 GPU in combination with pose prediction. For each target, DrugBLIP is executed with 10 different initial conformations and the total time consumption is recorded. In addition to traditional docking programs, we also compared against the recently proposed Boltz-2 Passaro et al. (2025). The results show that DrugBLIP achieves a speed hundreds of times faster than conventional docking tools, and remains substantially faster than Boltz-2 while maintaining competitive accuracy because Boltz-2 reports runtimes of ∼1–10 s per complex for the full global docking workflow.

Table 3. Comparison of efficiency of different methods

Method	QVINA-W	GNINA	SMINA	GLIDE	DrugBLIP
Time(s)	49	247	146	1405	0.2

References

H. M. Berman, J. Westbrook, Z. Feng, G. Gilliland, T. N. Bhat, H. Weissig, I. N. Shindyalov, and P. E. Bourne. The protein data bank. *Nucleic acids research*, 28(1):235–242, 2000.

B. Gao, B. Qiang, H. Tan, Y. Jia, M. Ren, M. Lu, J. Liu, W.-Y. Ma, and Y. Lan. Drugclip: Contrasive protein-molecule representation learning for virtual screening. *Advances in Neural Information Processing Systems*, 36, 2024.

T. A. Halgren. Merck molecular force field. i. basis, form, scope, parameterization, and performance of mmff94. *Journal of computational chemistry*, 17(5-6):490–519, 1996.

D. P. Kingma and J. Ba. Adam: A method for stochastic optimization. In *ICLR (Poster)*, 2015. URL `http://arxiv.org/abs/1412.6980`.

G. Landrum et al. Rdkit: A software suite for cheminformatics, computational chemistry, and predictive modeling. *Greg Landrum*, 8(31.10):5281, 2013.

V. Le Guilloux, P. Schmidtke, and P. Tuffery. Fpocket: an open source platform for ligand pocket detection. *BMC bioinformatics*, 10:1–11, 2009.

S. Passaro, G. Corso, J. Wohlwend, M. Reveiz, S. Thaler, V. R. Somnath, N. Getz, T. Portnoi, J. Roy, H. Stark, et al. Boltz-2: Towards accurate and efficient binding affinity prediction. *BioRxiv*, pages 2025–06, 2025.

S. Riniker and G. A. Landrum. Better informed distance geometry: using what we know to improve conformation generation. *Journal of chemical information and modeling*, 55(12):2562–2574, 2015.

V. G. Satorras, E. Hoogeboom, and M. Welling. E (n) equivariant graph neural networks. In *International conference on machine learning*, pages 9323–9332. PMLR, 2021.

G. Zhou, Z. Gao, Q. Ding, H. Zheng, H. Xu, Z. Wei, L. Zhang, and G. Ke. Uni-mol: A universal 3d molecular representation learning framework. In *The Eleventh International Conference on Learning Representations*, 2023.
